# Supplementary material for: Recovery of proofreading-impaired SARS-CoV-2 reveals a mutator phenotype and an ExoN activity threshold for viability
Source: J Virol. 2026 Jul 29;100(8):e00809-26. doi: 10.1128/jvi.00809-26 (PMC13483246; doi:10.1128/jvi.00809-26)
Supplement: Supplementary file 1 [file jvi.00809-26-s0004.docx]

**Supplemental Table 1. Oligonucleotides used in this study.**

| **Oligo Name** | **Sequence (5’-3’)** |
| --- | --- |
| **En passant mutagenesis** | |
| NSP14(K9A)-FW | TGGCAACTTTACAAGCTGAAAATGTAACAGGACTTTTTGCAGATTGTAGTAAGGTAATCACAGGATGACGACGATAAGTAGGG |
| NSP14(K9A)-RV | TGTGTAGGATGTAACCCAGTGATTACCTTACTACAATCTGCAAAAAGTCCTGTTACATTTTCAACCAATTAACCAATTCTGATTAG |
| NSP14(K61A)-FW | GGACATGACCTATAGAAGACTCATCTCTATGATGGGTTTTGCAATGAATTATCAAGTTAATGGAGGATGACGACGATAAGTAGGG |
| NSP14(K61A)-RV | TGATAAACATGTTAGGGTAACCATTAACTTGATAATTCATTGCAAAACCCATCATAGAGATGACAACCAATTAACCAATTCTGATTAG |
| NSP14(K139A)-FW | TATGTTGATACACCTAATAATACAGATTTTTCCAGAGTTAGTGCTGCACCACCGCCTGGAGATCAATTAGGATGACGACGATAAGTAGGG |
| NSP14(K139A)-RV | GTACATAAGTGGTATGAGGTGTTTAAATTGATCTCCAGGCGGTGGTGCAGCACTAACTCTGGAAAAATCAACCAATTAACCAATTCTGATTAG |
| NSP14(K9E)-FW | CAACTTTACAAGCTGAAAATGTAACAGGACTTTTTGAAGATTGTAGTAAGGTAATCACTGAGGATGACGACGATAAGTAGGG |
| NSP14(K9E)-RV | GTAGGATGTAACCCAGTGATTACCTTACTACAATCTTCAAAAAGTCCTGTTACATTTTCACAACCAATTAACCAATTCTGATTAG |
| NSP14(D90A-E92A)-FW | AAGAAGCTATAAGACATGTACGTGCATGGATTGGCTTCGCAGTCGCAGGGTGTCATGCTACTAGAGAAGGATGACGACGATAAGTAGGG |
| NSP14(D90A-E92A)-RV | ATTGGTACCAACAGCTTCTCTAGTAGCATGACACCCTGCGACTGCGAAGCCAATCCATGCACGTACAACCAATTAACCAATTCTGATTAG |
